# Precourse Preparation Using a Serious Smartphone Game on Advanced Life Support Knowledge and Skills: Randomized Controlled Trial

**DOI:** 10.2196/16987

**Published:** 2020-03-09

**Authors:** Pariwat Phungoen, Songwoot Promto, Sivit Chanthawatthanarak, Sawitree Maneepong, Korakot Apiratwarakul, Praew Kotruchin, Thapanawong Mitsungnern

**Affiliations:** 1 Department of Emergency Medicine Faculty of Medicine Khon Kaen University Khon Kaen Thailand; 2 CPR Training Unit, Srinagarind Hospital Faculty of Medicine Khon Kaen University Khon Kaen Thailand

**Keywords:** CPR training, gamified learning, medical education, serious game learning

## Abstract

**Background:**

In the past several years, gamified learning has been growing in popularity in various medical educational contexts including cardiopulmonary resuscitation (CPR) training. Furthermore, prior work in Basic Life Support (BLS) training has demonstrated the benefits of serious games as a method for pretraining among medical students. However, there is little evidence to support these benefits with regard to Advanced Life Support (ALS) training.

**Objective:**

We compare the effects of a brief precourse ALS preparation using a serious smartphone game on student knowledge, skills, and perceptions in this area with those of conventional ALS training alone.

**Methods:**

A serious game (Resus Days) was developed by a Thai physician based on global ALS clinical practice guidelines. Fifth-year medical students were enrolled and randomized to either the game group or the control group. Participants in both groups attended a traditional ALS lecture, but the game group was assigned to play Resus Days for 1 hour before attending the lecture and were allowed to play as much as they wished during the training course. All students underwent conventional ALS training, and their abilities were evaluated using multiple-choice questions and with hands-on practice on a mannequin. Subject attitudes and perceptions about the game were evaluated using a questionnaire.

**Results:**

A total of 105 students participated in the study and were randomly assigned to either the game group (n=52) or the control group (n=53). Students in the game group performed better on the ALS algorithm knowledge posttest than those in the control group (17.22 [SD 1.93] vs 16.60 [SD 1.97], *P*=.01; adjusted mean difference [AMD] 0.93; 95% CI 0.21-1.66). The game group’s pass rate on the skill test was also higher but not to a statistically significant extent (79% vs 66%, *P*=.09; adjusted odds ratio [AOR] 2.22; 95% CI 0.89-5.51). Students indicated high satisfaction with the game (9.02 [SD 1.11] out of 10).

**Conclusions:**

Engaging in game-based preparation prior to an ALS training course resulted in better algorithm knowledge scores for medical students than attending the course alone.

**Trial Registration:**

Thai Clinical Trials Registry HE611533; https://tinyurl.com/wmbp3q7

## Introduction

Advanced Life Support (ALS), widely accepted as the gold standard of care in patients with cardiopulmonary arrest, requires specialized skills and knowledge [1,2]. Training in ALS is thus recommended for health care providers who are likely to encounter either in-hospital or out-of-hospital cardiac arrest (OHCA) [2,3]. The ALS training course covers the knowledge, skills, and attitudes required for managing both cardiac arrest and periarrest problems. It includes lecture-based teaching, workshops, skill stations, simulation-based training with high-fidelity mannequins, and debriefing [4-6]. In order to maximize learning outcomes, the current American Heart Association guidelines for cardiopulmonary resuscitation (CPR) recommend students prepare in advance for the ALS training course [7]. This can be addressed in a variety of ways including the provision of precourse reading (CD-based, e-learning–based, etc), online and precourse testing, and opportunities to practice pertinent technical skills [6]. However, there has been no evidence demonstrating the benefits of precourse training when taken in conjunction with a conventional ALS training program [7-10].

Over the past several years, gamified learning has been growing in popularity in various medical educational contexts including CPR training [11-20]. The benefits of this learning method are that it improves learning outcomes by creating a high level of engagement among participants, facilitating learners’ holistic understanding of scientific concepts, and providing flexible learning methods and real-time feedback [18,21-23]. Recent studies have found that gamified learning can improve medical knowledge, skills, attitudes, and satisfaction when compared with traditional education methods [12,20,24]. Furthermore, prior work in Basic Life Support (BLS) training has demonstrated the benefits of serious games as a method for pretraining among medical students [18]. However, there is little evidence to support these benefits with regard to ALS training, with only one study reporting that game-based training augments retention of acquired skills and knowledge [13].

**Figure 1:**
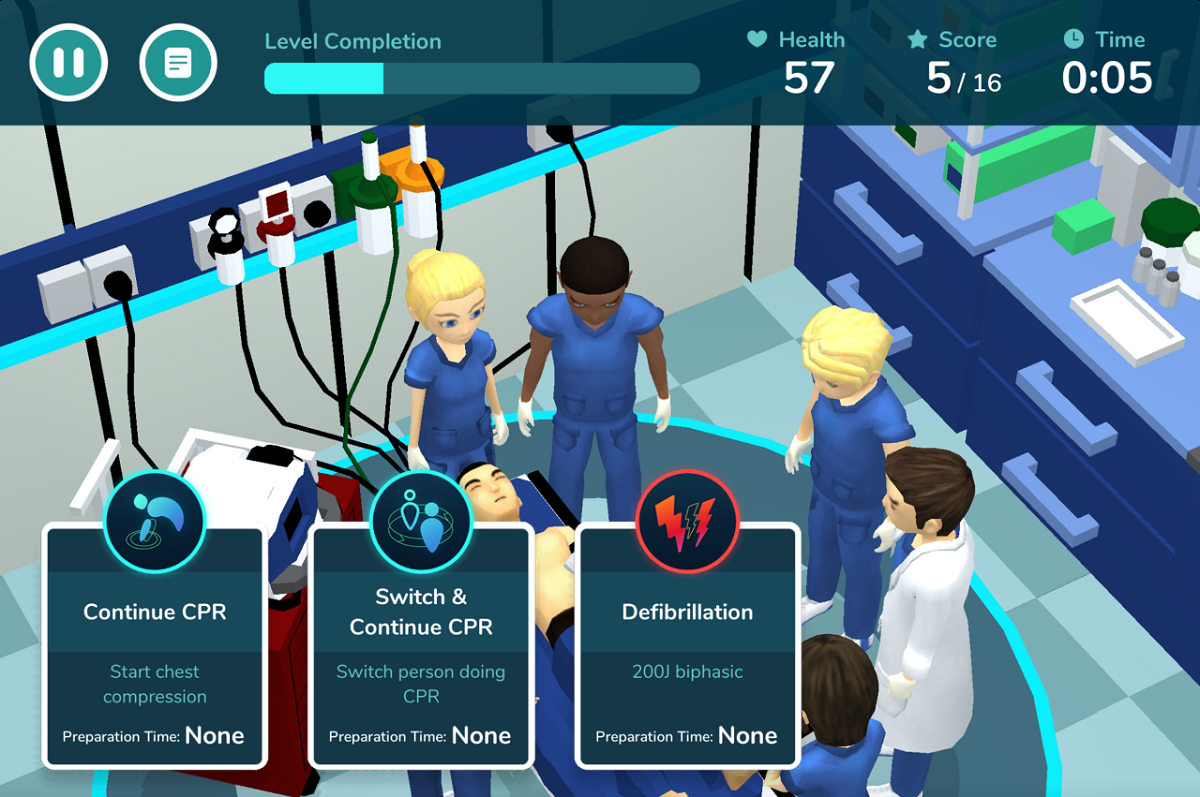
Screenshot from Resus Days.

Resus Days is a serious smartphone game developed by a Thai physician using standard ALS guidelines and is available online (Figure 1) [25]. The aim of the game is to familiarize students with the ALS algorithms for cardiac arrest and periarrest scenarios, leading to shorter knowledge acquisition time. The objective of this study was to evaluate the effects of precourse preparation using this game on students’ ALS knowledge, skills, and perceptions. We hypothesized that adding this game to the traditional precourse training would lead to higher ALS knowledge scores.

## Methods

### Study Design

This was a prospective, randomized controlled parallel group trial in a tertiary university hospital in Thailand. It was approved by the Khon Kaen University institutional committee for ethics in human research and registered with the Thai Clinical Trials Registry [HE611533]. Written informed consent was obtained from each participant prior to enrollment.

### Participants and Randomization

Participants were fifth-year medical students at the Khon Kaen University Faculty of Medicine who attended the ALS training course offered by the faculty’s CPR unit in April 2019. Eligibility criteria included not having participated in any type of ALS course in the past and never having played Resus Days. After enrollment, participants were randomly assigned to one of two groups (control group or game group) at a 1:1 allocation ratio based on a computer-generated random number list [26].

### Sample Size Calculation

The sample size for analysis of covariance of two levels and two covariates was determined based on our pilot data using power analysis. We hypothesized that the difference in knowledge between the two groups was approximately 5%, and that there would be moderate consistency between pretest and posttest scores. The power analysis was determined using an alpha of .05 and power of 0.80. This resulted in an estimated desired effect sample size of at least 22 subjects in each group.

### Study Interventions

Prior to any intervention, we tested students’ baseline ALS knowledge using a multiple-choice pretest. Students in the game group then played Resus Days using their own smartphone for 1 hour in normal mode. After this, students in both groups attended an ALS training course taught by CPR instructors. Students in the game group were allowed to play Resus Days as much as they wished during the 2-day ALS training course.

### Game Description

Resus Days was developed by Rath Panyowat. It is a mobile simulation game that allows health care professionals to practice CPR through repetitive playing. Players must resuscitate a simulated patient in various scenarios by choosing treatment methods based on the ALS algorithm until the patient’s cardiac rhythm becomes normal. There are two game modes: normal and physician. In normal mode, a player receives hints regarding diagnosis and treatment and gains points if the prescribed treatment is correct. In physician mode, there are no hints and points are subtracted if the prescribed treatment is incorrect. There are a total of 7 scenarios including cardiac arrest, bradycardia, tachycardia, and several simulated megacode (mixed algorithm) scenarios. To win in each scenario, the player must strictly follow the ALS algorithm within the time allotted (Figure 1).

### Advanced Life Support Training Course

The ALS training course was held over 2 days. The first day of the program consisted of approximately 6 hours of lectures, and the second day consisted of approximately 8 hours of hands-on workshops, skill station activities, simulation-based training with high-fidelity ALS simulator mannequins with the SimPad system (Laerdal Medical). The student-to-instructor ratio was 1:6. At the end of the course, student ALS knowledge was reassessed using multiple-choice questions in accordance with ALS guidelines, and their practical skills were evaluated individually by examiners blinded to group allocation. We used a questionnaire to assess student attitudes and perceptions regarding both the course and the game.

### Assessment of Learning Outcomes, Attitudes, and Perceptions

#### Knowledge Test

The knowledge test was divided into two parts: one to assess knowledge of the ALS algorithm and another to assess general ALS knowledge. The first part consisted of 20 multiple-choice questions, each with 5 possible answers, and the second consisted of 30 multiple-choice questions. The ALS algorithm test focused on management of life-threatening conditions (using the ALS adult cardiac arrest algorithm) and symptomatic bradycardia and tachycardia. The general ALS knowledge test focused on BLS knowledge, general ALS knowledge, and postresuscitation care (see Multimedia Appendix 1).

#### Advanced Life Support Skill Tests

Each student was given a series of three different 5-minute megacode (mixed algorithm) simulated ALS scenarios (chosen randomly using the closed envelope method), and their performance was evaluated by certified ALS course providers (see Multimedia Appendix 2 for details regarding each scenario). Students passed the skill tests if they had scores of 80% or greater and no critical errors.

#### Questionnaire Regarding Participant Attitudes and Perceptions

We asked students to rate their attitudes and perceptions regarding the game on a scale of 1 to 10 (1=disagree completely and 10=agree completely). An open-ended question was used to assess any problems students had while playing the game, opinions about the game, and recommendations for further improvement (see Multimedia Appendix 3).

### Game Score

Students received a score from 0 (if the patient died) to 3 (if patient survived and the most appropriate treatments were prescribed) for each scenario. The highest possible overall score was 21 points (see Multimedia Appendix 4).

### Statistical Analysis

Categorical variables were expressed as frequencies and percentages. Continuous data were expressed as means and standard deviations. Differences between groups in terms of baseline characteristics were compared using an independent sample *t* test. An analysis of covariance model was used to compare posttest scores between the two groups adjusted for baseline score measurements. The Pearson chi-square test was used to compare the skill-test pass rate and other binary variables. The correlation between game scores before ALS training and knowledge pretest scores and between game scores post-ALS training and knowledge posttest scores were analyzed using Pearson correlation coefficients. All data analyses were performed using Stata version 10 (StataCorp LLC).

## Results

**Figure 2:**
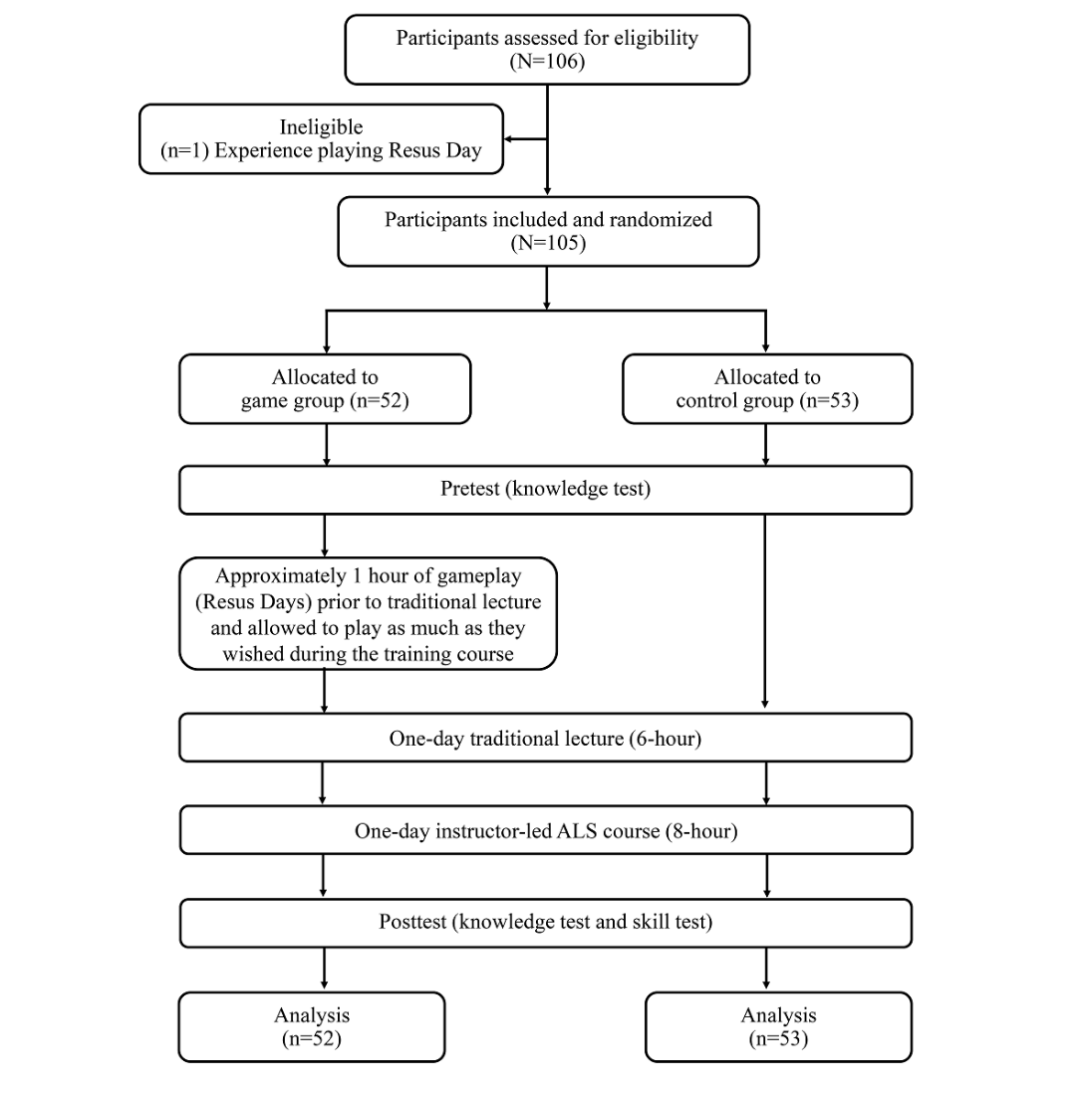
Flow diagram of participant selection.

A total of 105 students participated in the study and were randomly assigned to either the game group (n=52) or the control group (n=53; Figure 2). All students completed the trial and were included in the data analysis. Demographic characteristics of the two study groups are shown in Table 1. There were no significant differences between the groups in terms of sex, age, grade point average, CPR experience, CPR confidence, or learning outcome scores.

**Table 1:** Participant demographics.

Characteristic			Game group (n=52)		Control group (n=53)		*P* value
Age in years, mean (SD)			23.06 (1.02)		23.02 (1.07)		.85
Male, n (%)			33 (63)		34 (64)		.94
GPA^a^, range 0.00-4.00, mean (SD)			3.33 (0.32)		3.33 (0.38)		.97
CPR^b^ member experience, n (%)			38 (73)		42 (79)		.61
**Knowledge pretest score, mean (SD)**							
	Algorithm knowledge test	7.19 (3.02)		8.17 (3.17)		.11	
	General knowledge test	12.54 (2.90)		12.79 (3.04)		.66	
Pretraining CPR confidence (1-10), mean (SD)			3.04 (2.06)		3.17 (2.27)		.86

^a^GPA: grade point average.

^b^CPR: cardiopulmonary respiration.

**Figure 3:**
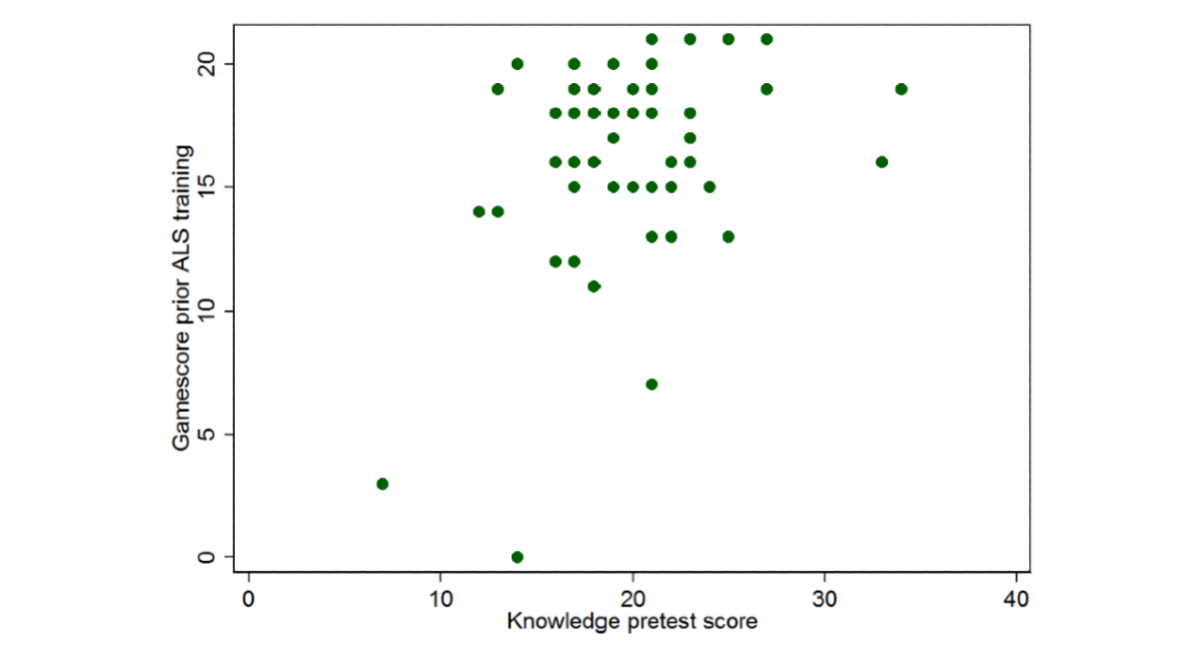
Correlation between game score before Advanced Life Support training and knowledge pretest score was low (*P*=.01, r=.37).

**Figure 4:**
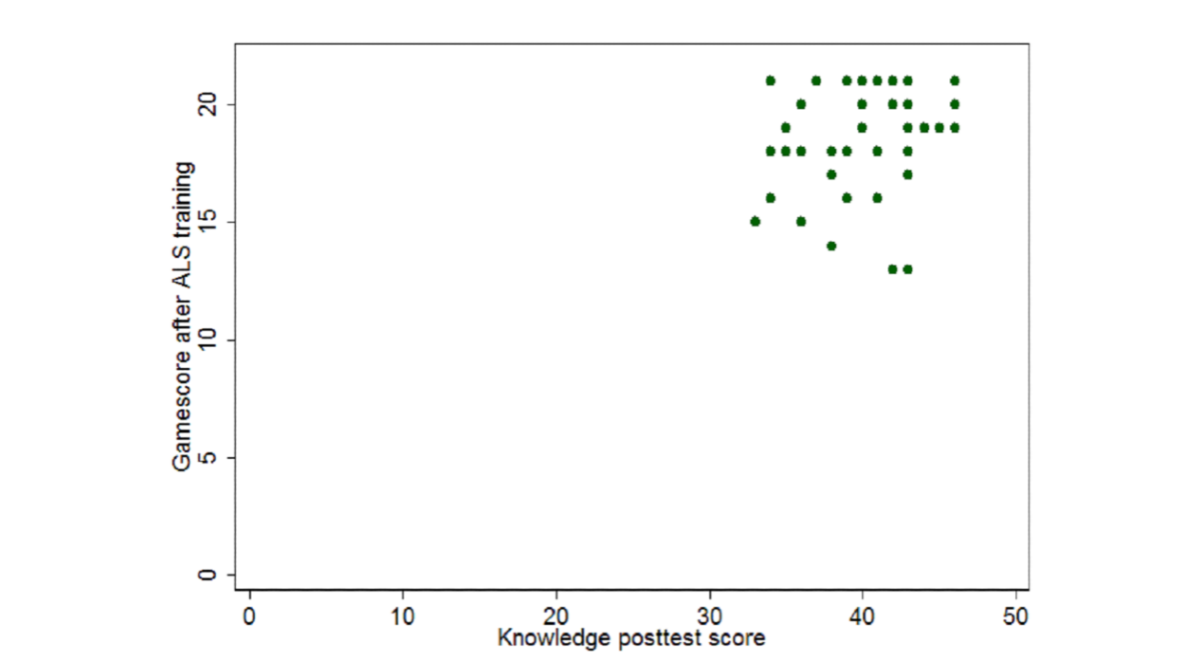
Correlation between game score post-Advanced Life Support training and knowledge posttest score was low (*P*=.07, r=.25).

Students in the game group performed better on the ALS algorithm knowledge posttest than those in the control group (17.22 [SD 1.93] vs 16.60 [SD 1.97], *P*=.01; adjusted mean difference [AMD] 0.93; 95% CI 0.21 to 1.66). They also had a higher pass rate on the skill test (79% vs 66%, *P*=.09; adjusted odds ratio [AOR] 2.22; 95% CI 0.89 to 5.51) and indicated greater confidence in performing CPR (7.87 [SD 1.05] vs 7.85 [SD 2.26], *P*=.51; 95% CI –0.45 to 0.92; Table 2). However, these differences were not statistically significant. Students also indicated high satisfaction with the game (9.02 [SD 1.11] out of 10). The correlations between pre- and posttraining game scores and knowledge scores are shown in Figures 3 and 4. There was little correlation between pretraining game and pretest scores (*P*=.01, *r*=.37; Figure 3).

Student attitudes and perceptions regarding Resus Days are summarized in Table 3. Most students were satisfied with the game and indicated that it helped them memorize the CPR algorithm and drug dosages and increased their confidence in making decisions.

**Table 2:** Comparison of learning outcomes between the game group and control group.

Characteristic		Game group (n=52)	Control group (n=53)	AMD^a^/AOR^b^ (95% CI)	*P* values
**Algorithm knowledge score, mean (SD)**					
	Posttest score^c^	17.33 (1.93)	16.60 (1.97)	0.93 (0.21 to 1.66)^a^	.01
	Score improvement (post-pre)	10.13 (3.00)	8.43 (3.10)	—	
**General knowledge score**					
	Posttest score^d^, mean (SD)	22.88 (2.49)	23.45 (2.89)	–1.69 (–6.11 to 2.72)^a^	.45
	Score improvement (post-pre), mean (SD)	10.35 (3.00)	10.66 (3.63)	—	
	ALS^e^ skill test pass^f^, n (%)	41 (79)	35 (66)	2.22 (0.89 to 5.51)^b^	.09
	CPR^g^ confidence^d^ (postcourse), mean (SD)	7.87 (1.05)	7.85 (2.26)	0.23 (–0.45 to 0.92)^a^	.51

^a^AMD: adjusted mean difference.

^b^AOR: adjusted odds ratio.

^c^Analysis of covariance model adjusted for baseline measurement.

^d^Analysis of covariance model adjusted for baseline measurement and interaction.

^e^ALS: Advanced Life Support.

^f^Binary logistic regression (adjusted for grade point average, sex, and knowledge pretest score).

^g^CPR: cardiopulmonary resuscitation.

**Table 3:** Participant attitudes and perceptions regarding Resus Days based on a score of 1=strongly disagree to 10=strongly agree.

Attitudes and perceptions		Participant in game group (n=52)
Overall satisfaction with Resus Days, median (IQR^a^)		9 (8-10)
Resus Days helped improve my CPR^a^ knowledge, median (IQR)		9 (8-10)
**If you think game helped improve your ALS^a^ knowledge, please specify a reason (students could select more than one answer; n=62), n(%)**		
	The game helped me become familiar with cardiac arrest scenarios	15 (24)
	The game emphasized critical points in the ALS algorithm	13 (21)
	Repeatedly playing the game helped me memorized the CPR algorithm and correct medication dosages	10 (16)
	The game improved my decision-making ability and made me more confident	7 (11)
	Other	17 (27)
**Suggestions for improving the game, n** **(%)**		
	Create more scenarios (eg, postcardiac arrest care, myocardial infarction)	22 (42)
	Make changes with regard to gameplay	6 (12)
	Allow the player to choose the drug doses	5 (10)
	Bug fixes	2 (4)
	Other	4 (8)
	None	13 (25)
**Frequency of playing any other online games, n** **(%)**		
	1 day per week	14 (27)
	Every day	12 (23)
	<1 day per week	11 (21)
	Every 2 to 3 days	8 (15)
	Never	7 (14)

^a^IQR: interquartile range.

^b^CPR: cardiopulmonary resuscitation.

^c^ALS: Advanced Life Support.

## Discussion

### Principal Findings

ALS certification is mandatory for all medical students entering their clinical years. This study demonstrates the merits of using a serious video game to augment ALS knowledge, especially regarding the ALS algorithm. In addition, the game group had a higher pass rate on the ALS skill test and indicated greater satisfaction with their training.

Previous studies have also examine the effectiveness of games in helping students practice their resuscitation skills. Creutzfeldt et al [18], for example, conducted a study that evaluated the efficacy of game technology as part of a pre-BLS training program for medical students, which led to improved BLS knowledge and performance. These improvements were associated with adherence to the guidelines, which resulted in students performing chest compressions with the appropriate frequency. However, that study had a limited sample size, and its findings required confirmation. Our findings are consistent with those of Creutzfeldt et al [18], but we had a larger sample size and applied game-based learning to training in ALS, which has a more complicated algorithm. The game group exhibited clear improvement with regard to ALS algorithm knowledge. They also demonstrated improvements in terms of skill, but not to a statistically significant extent.

Several studies have reported beneficial effects of game-based training on medical student learning outcomes [18,21-23]. A large number of the medical students in our study had positive attitudes about Resus Days, indicating that the game helped them become more familiar with various cardiac arrest scenarios prior to the ALS training course. These findings are in line with those of a study by Cheng et al [22], which found that game-based learning helped learners develop a holistic understanding of scientific concepts. Furthermore, some students stated that playing the game helped them memorize the CPR algorithm and medications. This may be due to improvements in cognitive function as a result of repetitive playing. Step-by-step game playing, in which scenarios become increasingly complicated, has been shown to stimulate critical thinking and increase engagement [18,21].

Another interesting finding was the low correlation between student game scores and their knowledge scores. This can be explained by the fact that some students may not have been familiar with the game mechanics at first, resulting in them getting low scores despite being knowledgeable about ALS and having good pretest or posttest scores. Because of this, we do not recommend using a player’s game score as a substitute for traditional testing. However, regardless of players’ scores, their knowledge was significantly improved by playing the game. This is partially because the game provided the correct answers at the end of each scenario even if the player failed.

Several methods have been proposed to prepare trainees before attending ALS training courses. We found that engaging in game-based preparation prior to precourse ALS lectures (consisting of precourse algorithm rehearsal and lectures accompanied by Microsoft PowerPoint slides or lecture notes) was more beneficial than conventional precourse lectures alone. Although it was only an add-on intervention, the observed benefits demonstrated the ability of gamified training to improve a complicated medical course.

Most of the studies that have demonstrated the benefits of game-based learning have been conducted in high-income countries [20]. This study, however, was conducted in a low-middle income country and found that, even in this setting, game-based learning was practical and effective. In the near future, this may be used to help train large numbers of medical students or personnel in remote areas who have little or no access to traditional classroom-based lectures. However, if game-based learning is to replace conventional ALS lectures, a randomized controlled trial using a well-designed game may be necessary.

### Strengths and Limitations

One strength of our study is that it is the first to demonstrate the effectiveness of ALS pretraining using a smartphone game and addresses the feasibility of gamified learning in training students to treat highly complicated medical conditions. Second, we used a game that is available in an online store and therefore available to anyone with access to a compatible device. Although previous studies have developed their own games as tools for research [15,17,19], the initial design of such a study is costly and time consuming. Moreover, using a custom game specifically developed for a particular study would limit its generalizability. Third, as our study was a randomized controlled trial, differences in baseline characteristics between students in the game and control groups were limited. Finally, we performed a comparison of gamified learning with a standard ALS training course that was provided by certified CPR providers and that met international standards.

However, our study also had some potential limitations. First, this was a single-center study, which limits the generalizability of the results. Second, we evaluated students based on their knowledge scores, which are imperfect indicators of how someone will perform in real-world scenarios. Third, the sample size was only large enough to evaluate student knowledge scores and not their results on the ALS skill test. Fourth, most of the participants in this study were medical students who were familiar with smartphone games. It is unclear how effective this kind of gamified learning would be in an older population such as senior doctors. Fifth, the students in the game group were assigned to play Resus Days for 1 hour before attending the lecture and were allowed to play as much as they wished during the training course; however, we did not have specific protocol and did not collect any data regarding the frequency of gameplay during the 2-day training. Finally, the content in this game was developed by an individual physician based on standard guidelines, not by a content expert or trusted organization.

### Conclusions

Our results demonstrate that incorporating a serious smartphone game (Resus Days) in medical students’ ALS training leads to better algorithm knowledge scores than conventional training alone. In addition, students in the game group indicated high satisfaction with their training. Although there was no significant difference between the two groups in terms of their pass rates on the ALS skills test, those who trained using the serious game tended to do better.

## Appendix

Multimedia Appendix 1 Knowledge test.

Multimedia Appendix 2 Skill tests.

Multimedia Appendix 3 Questionnaire given to intervention group (adapted from original questionnaire in Thai language).

Multimedia Appendix 4 Resus Days screenshots.

Multimedia Appendix 5 CONSORT-EHEALTH checklist (V1.6.1).

## Abbreviations

ALS: Advanced Life Support
AMD: adjusted mean difference
AOR: adjusted odds ratio
BLS: Basic Life Support
CPR: cardiopulmonary resuscitation
OHCA: out-of-hospital cardiac arrest
