# Supplementary material for: Precourse Preparation Using a Serious Smartphone Game on Advanced Life Support Knowledge and Skills: Randomized Controlled Trial
Source: J Med Internet Res. 2020 Mar 9;22(3):e16987. doi: 10.2196/16987 (PMC7091031; doi:10.2196/16987)
Supplement: Multimedia Appendix 1 [file jmir_v22i3e16987_app1.pdf]

Multimedia appendix 1: Knowledge test

Advanced life support exam

Part-1 Algorithm knowledge test

Mega code scenario I (Answer question 1-5)

1. Scenario data: An 82-year-old female in your ER complains of weakness, lightheadedness, and mild pain in her chest. You are assessing her at this time.
   Assessment:
   Skin: Pale/Cool/Cyanotic
   Cardio: Weak radial pulse, HR 32, BP 70/30
   Resp: Clear in all lung fields, RR 22, oxygen sat 88%
   CNS: Weak but responds to questions, oriented
   Monitor: (see below)

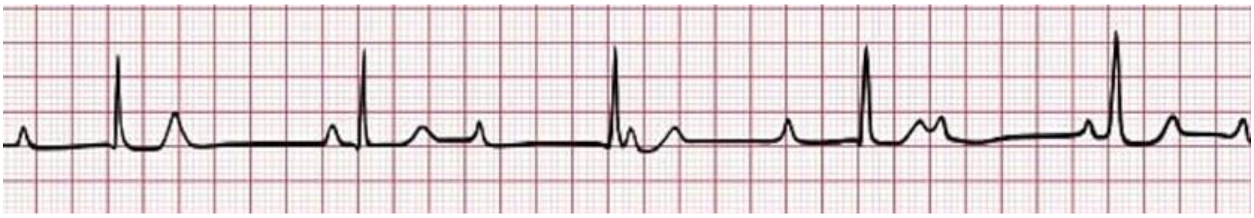

Based on the assessment what will be your next step?

A. Maintain airway, breathing and circulation
B. establish IV
C. attach ECG and monitor
D. provide oxygen
E. all of the above

2. Based on the assessment (on question number 41), what will be your next intervention?
   A. Adrenaline 0.3-0.5 mg IM
   B. Prepare for transcutaneous pacing
   C. Atropine 0.5 mg IV push
   D. Epinephrine 2-10 mcg per min
   E. Give 1 mg atropine IV push

3. Five minute later, you recognize that the patient’ s condition is worsening so you begin pacing. Your attempts to pace the patient fail, and she becomes unresponsive. She has no pulse and you see this rhythm change. What is your first intervention?

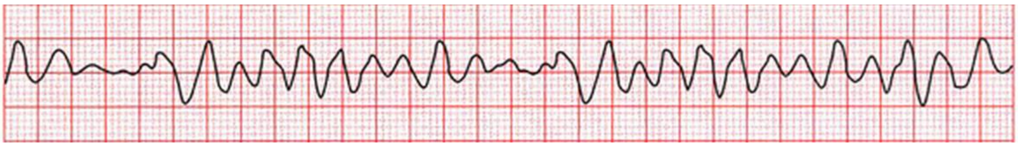

A. give epinephrine 1mg IV push

B. CPR 5 cycle

C. give 1 mg atropine IV push

D. give 1 unsynchronized shock (Defibrillation)

E. give 1 synchronized shock 120-200 J

4. Following question number 43, the patient remains no rhythms change after 2 min of CPR and has no pulse. What is your next intervention?

A. deliver 1 shock followed by amiodarone 300 mg IV

B. deliver 1 shock followed by 5 cycles CPR

C. give amiodarone 300 mg IV x 1

D. give epinephrine 1 mg IV

E. give 1 synchronized shock 120-200 J

5. After you do intervention, you follow the epinephrine with completion of 5 cycles of CPR, a rhythm checks and a shock delivery (200 J) since there was no rhythm change. You begin another cycle of CPR and want to give an antiarrhythmic. Amiodarone is not available. What is your other choice?

A. Labetalol

B. Lidocaine

C. Dopamine

D. Cardizem

E. Procanamide

Mega code scenario II (answer question 6-10)

6. 50-year-old man brought to the ER by his son. He complains of occasional dizziness and lightheadedness for about 12 hours. He says that he is also occasionally short of air. The assessment is as follows:

    Skin: Warm/Dry

    Cardiovascular: Pulse palpable (strong), BP 106/68, HR 140's; no chest pain

    Respiratory: Lungs clear both side, Pulse Oximetry 96% ; RR 20

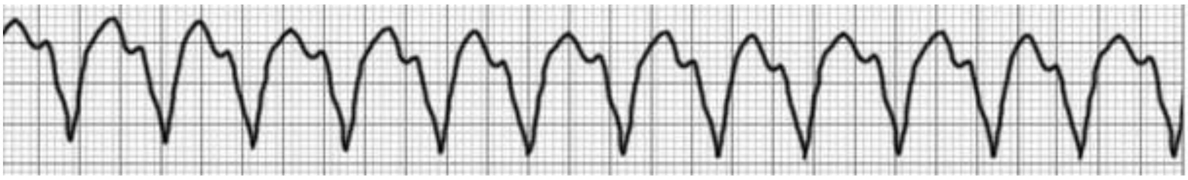

    A. stable ventricular tachycardia
    B. unstable ventricular tachycardia
    C. ventricular fibrillation
    D. Stable supraventricular tachycardia
    E. unstable supraventricular tachycardia

7. Following Megacode scenario I, the patient should now be prepared to be given which treatment?
    A. Overdrive pacing rate 120 b/min
    B. Adenosine 12 mg IV push
    C. Adenosine 6 mg IV push or Amiodarone 150 mg over 10 min IV drip
    D. Amiodarone 300 mg IV push
    E. Synchronized cardioversion at 100 J

8. While prepare for medication, your patient begins to experience severe chest pain and becomes short of air. His vital signs also change (BP 70/30; HR 160; RR 28). The patient’s rhythm on the monitor now looks like this:

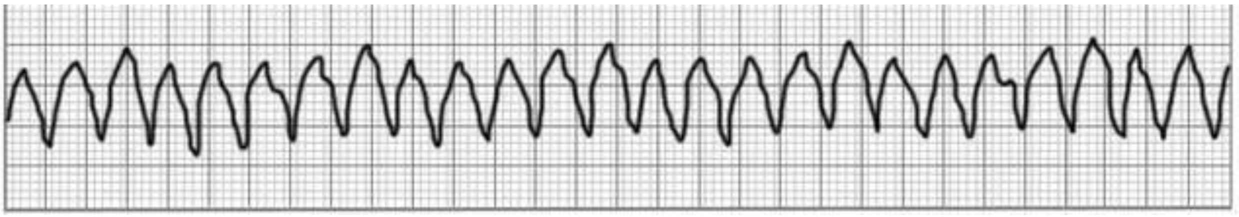

    The patient still has a pulse. What should be your intervention at this time?
    A. Immediate defibrillation 120 J.
    B. Perform immediate synchronized cardioversion 50 J

C. Perform immediate synchronized cardioversion 100 J

D. Give epinephrine 1mg IV/IO

E. CPR 5 cycle

9. After treatment, the pt. deteriorates further and loses consciousness. His rhythm changes and the patient has no pulse. This is what you see on the monitor:

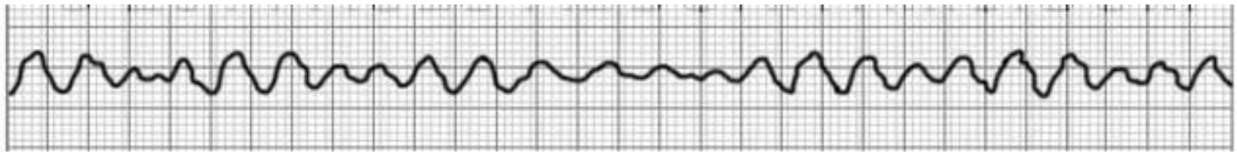

What is your first intervention?

A. give 1 unsynchronized shock (Defibrillation)

B. give epinephrine 1mg IV push

C. CPR 5 cycle

D. give 1 mg atropine IV push

E. give 1 synchronized shock 120-200 J

10. You resume 5 cycles of CPR and you given the patient the epinephrine. Identify the correct dose and frequency below

A. epinephrine 1 mg IV x 2 doses

B. epinephrine 0.5 mg IV the every 3-5 minutes

C. epinephrine 0.5 mg IM the every 3-5 minutes

D. epinephrine 1 mg IV the every 3-5 minutes

E. epinephrine 2 mg IV the every 3-5 minutes

11. You are evaluating a 70-year-old man with sudden palpitation. The patient's blood pressure is 130/59 mm Hg, the heart rate is 170/min, the respiratory rate is 16 breaths/min, and the pulse oximetry reading is 96%. The lead II ECG is displayed below. <u>What is the next step of management?</u>

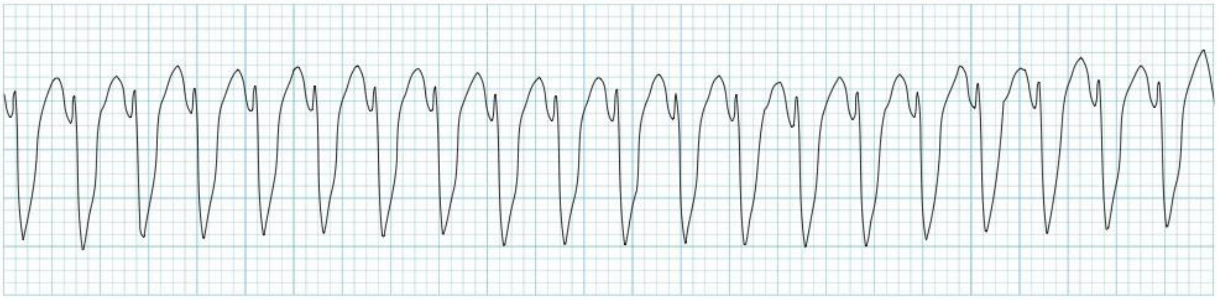

A. Adenosine 6 mg IV push

B. Adenosine 12 mg IV push

C. Amiodarone 150 mg over 10 min IV push

D. Amiodarone 300 mg IV push

E. Synchronized cardioversion at 100 J

12. The decision point in the bradycardia algorithm (stable or unstable) is the determination of:

A. Adequate perfusion

B. Heart rate

C. Rhythm

D. All of above

E. None of above

13. What is the first line drug to treat symptomatic bradycardia?

A. Atropine 0.5 mg IV every 3-5 minutes

B. Atropine 1.0 mg IV every 3-5 minutes

C. Adrenaline 0.3-0.5 mg IM

D. Dopamine 2-20 mcg/kg/min

E. Epinephrine 2-10 mcg per min

14. You are evaluating a 80-year-old man with alteration of conscious. The patient is pale, diaphoretic, cool to the touch, and the pulse oximeter is 85%. The lead II ECG is displays below. For your first intervention, you give 0.5 mg IV bolus of atropine. The patient's condition does not change. <u>Based on the assessment what will be your next intervention?</u>

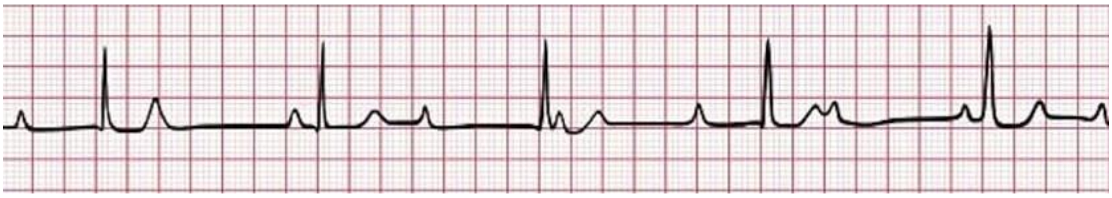

A. Dopamine IV infusion

B. Epinephrine IV infusion

C. Transcutaneous pacing

D. give adenosine 6mg rapid IV push

E. A or B or C

15. A 53-year-old man has palpitation. The patient's blood pressure is 102/59 mm Hg, the heart rate is 230/min, the respiratory rate is 16 breaths/min, and the pulse oximetry

reading is 96%. The lead II ECG is displayed below. A patient peripheral IV is in place. What is the next action?

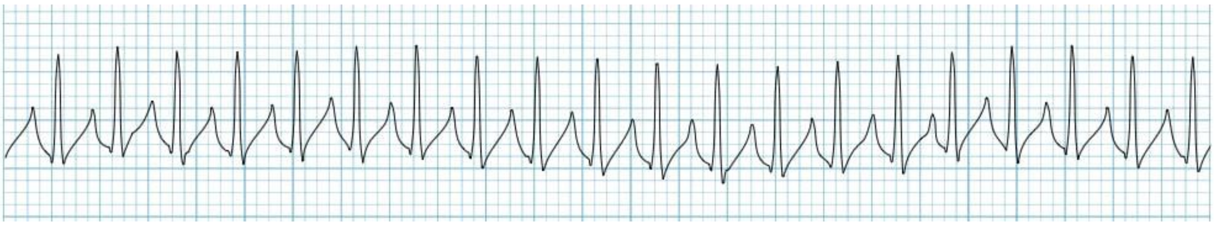

A. Procedural sedation

B. Synchronized cardioversion 50 J.

C. Adenosine 6 mg IV push

D. Adenosine 12 mg IV push

E. Vagal maneuvers

16. What is the recommended energy dose for biphasic synchronized cardioversion of atrial fibrillation?

A. 50 to 75 J

B. 75 to 100 J

C. 100 to 150 J

D. 120 to 200 J

E. 200 J

17. You are evaluating a 48-year-old man with crushing substernal chest pain. The patient is pale, diaphoretic, cool to the touch, and slow to respiratory rate is 18 breaths/min, and the pulse oximeter is unable to obtain a reading because there is no radial pulse. The lead II ECG displays a regular wide-complex tachycardia. What is the next step of management?

A. Amiodarone administration

B. Procedural sedation

C. Synchronized cardioversion 50 J

D. Synchronized cardioversion 100 J

E. Atropine administration

18. A monitored patient in the ICU developed a sudden onset of narrow-complex tachycardia at a rate of 220/min. the patient's blood pressure is 128/58 mm Hg, the $PETCO_2$ is 38 mm Hg, and the pulse oximetry reading is 98%. There is vascular access in the left arm, and the patient has not been given any vasoactive drugs. **A 12-lead ECG confirms a supraventricular tachycardia with no evidence of ischemia or**

infarction. The heart rate has not responded to vagal maneuvers. What is the next recommended intervention?

A. Adenosine 6 mg IV push

B. Adenosine 12 mg IV push

C. Amiodarone 300 mg IV push

D. Synchronized cardioversion at 50 J

E. Synchronized cardioversion at 200 J

19. The treatment sequence for bradycardia with poor perfusion is?

A. Maintain airway-breathing-circulation → consider transcutaneous pacing → administrator atropine → consider transvenous pacing

B. Maintain airway-breathing-circulation → administrator atropine → consider transcutaneous pacing → consider transvenous pacing

C. Administrator atropine → maintain airway-breathing-circulation → transcutaneous pacing → consider transvenous pacing

D. Administrator atropine → consider transcutaneous pacing → consider transvenous pacing → maintain airway-breathing-circulation

E. consider transcutaneous pacing → consider transvenous pacing → administrator atropine → maintain airway-breathing-circulation

20. For transcutaneous pacing, the current milliamperes (mA) output should be:

A. Set at 30-50 mA

B. Set at 50-100 mA

C. Set at the same capture dose

D. Set 2 mA or 10% above capture dose

E. Set 10 mA above capture dose

<u>Part-2 General knowledge test</u>

1. Examples of **advanced** airway adjuncts include all the following **except**:
   A. Oropharyngeal airway
   B. Laryngeal tube
   C. Laryngeal mask airway
   D. Combitube
   E. Endotracheal tube

2. Which one of the following identifies, in the correct order, the 4 step “universal step” required to operate an AED?
   A. Attach AED pads, power on AED, analyze rhythm, clear patient and deliver the shock if indicated
   B. Power on AED, attach AED pads, analyze rhythm, clear patient and deliver the shock if indicated
   C. Power on AED, analyze rhythm, attach AED pads, clear patient and deliver the shock if indicated
   D. Attach AED pads, analyze rhythm, power on AED, clear patient and deliver the shock if indicated
   E. None of above

3. Which action is included in the BLS Survey?
   A. Obtain IV/IO access
   B. Advanced airway management
   C. Rapid medication administration
   D. Early defibrillation
   E. Immediate transfer to intensive care

4. What is the initial priority for an unconscious patient with any tachycardia on the monitor?
   A. Review the patient’s home medications.
   B. Evaluate the breath sounds.
   C. Evaluation sign of unstable tachycardia
   D. Determine whether pulses are present.
   E. Administer sedative drugs.

5. You find an unresponsive patient who is not breathing. After activating the emergency response system, you determine that there is no pulse. What is your next action?
   A. Open the airway with a head tilt-chin lift.
   B. Administer epinephrine at a dose of 1 mg/kg.
   C. Deliver 2 rescue breaths each over 1 second.
   D. Start chest compressions
   E. Check for oxygen saturation

6. What is the appropriate rate of chest compressions for an adult in cardiac arrest?
   A. >80/min
   B. >100/min
   C. Rate of 100-120/min
   D. Rate of 120-130/min
   E. Rate of 130-140/min

7. What action is recommended to help minimize interruptions in chest compressions during CPR?
   A. Continue CPR while charging the defibrillator.
   B. Perform pulse checks immediately after defibrillation.
   C. Administer IV medications only when delivering breaths.
   D. Continue to use an AED even after the arrival of a manual defibrillator.
   E. Do not perform advance airway when CPR

8. What is the minimum depth of chest compressions for an adult in cardiac arrest?
   A. 2.5 cm.
   B. 4 cm.
   C. 5 cm.
   D. 6 cm.
   E. 7 cm.

9. Your rescue team arrives to find a 59-year-old man lying on the kitchen floor. You determine that he is unresponsive and notice that he is taking agonal breaths. What is the next step in your assessment and management of this patient?
   A. Apply the AED.
   B. Check the patient’s pulse.
   C. Open the patient’s airway.
   D. Check for oxygen saturation

E. Start chest compressions

10. How often should the team leader switch chest compressors during a resuscitation attempt?

A. Every minute

B. Every 2 minute

C. Every 3 minute

D. Sooner if compressor fatigue

E. B and D

11. You have completed 2 minutes of CPR. The ECG monitor displays the lead II rhythm below, and the patient has no pulse. Another member of your team resumes chest compressions, and an IV is in place. What management step is your next priority?

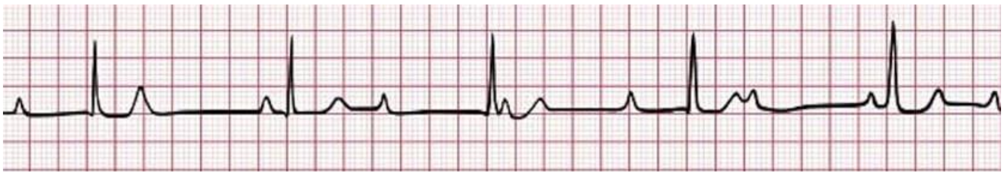

A. Give 0.5 mg of atropine.
B. Insert an advanced airway.
C. Administer a dopamine infusion.
D.Administer 1 mg of epinephrine.
E. Immediate shock 200 J.

12. If a patient remains in ventricular fibrillation despite 1 shock and 2 minutes of continuous CPR. The next intervention is to?

A. Administer amiodarone 300 mg IV push or Lidocaine.

B. Immediate resume CPR

C. Administer a second shock

D. Administer epinephrine 1 mg.

E. Insert an advanced airway.

13. You have begun CPR om an unresponsive 75-year old woman collapsed in cardiac arrest in the hospital emergency department waiting room. A nurse arrives and quickly attaches the AED, the first rhythm analysis indicates “no shock advised”. What is the most appropriate action to perform next?

A. Resume 5 cycles or 2 minutes of CPR

B. Unplug the connector from the machine

C. Ensure that no one, including you, is touching the person

D. Establish IV/IO access

E. Check the pad placement on the person's chest

14. What is the immediate danger of excessive ventilation during the post-cardiac arrest period for patients who achieve return of spontaneous circulation (ROSC)?
    A. Oxygen toxicity
    B. Pulmonary hypertension
    C. Cerebral edema
    D. Ventilation/perfusion mismatch
    E. Cerebral vasoconstriction.

15. What is the BEST strategy for performing high-quality CPR on a patient with an advanced airway in place?
    A. Provide compressions and ventilations with a 15:2 ratios.
    B. Provide compressions and ventilations with a 30:2 ratios.
    C. Provide a twice ventilation every 6 seconds during the compression pause.
    D. Provide ventilation every 4-5 second or 12-16 breath/min
    E. Provide continuous chest compressions without pauses and 10 ventilations per minute.

16. EMS personnel arrive to find a patient in cardiac arrest. Bystanders are performing CPR. After attaching a cardiac monitor, the responder observes the following rhythm strip. What is the most important early intervention?

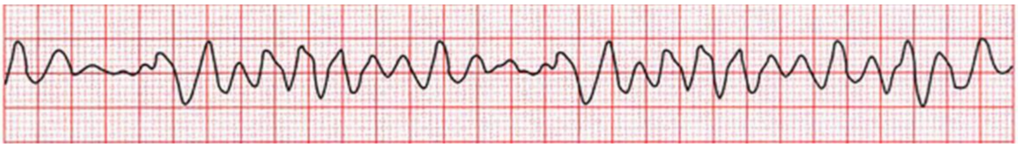

    A. Defibrillation
    B. Endotracheal intubation
    C. Epinephrine administration
    D. Antiarrhythmic ($MgSO_4$) administration
    E. Synchronized cardioversion

17. Three minutes after witnessing a cardiac arrest, one member of your-team inserts an endotracheal tube while another performs continuous chest compressions. During subsequent ventilation, you notice the presence of waveform on the capnography screen and $PETCO_2$ level of 8 mmHg. What is the significance of this finding?
    A. Chest compressions may not be effective.
    B. The endotracheal tube is no longer in the trachea.
    C. The patient meets the criteria for termination of efforts.

D. The team is ventilating the patient too often (hyperventilation).

E. The patient is return to spontaneous circulation

18. Which of the following actions, should you take to assess whether an ET tube is properly placed?

A. Make sure that compressions are not interrupted

B. Secured tube

C. Listen for bilateral breath sound

D. Check for adequate capillary refill

E. Present waveform in A-line monitors

19. Which finding is a sign of ineffective CPR?

A. PETCO2 > 10 mm Hg

B. Patient temperature >32°C

C. Diastolic intra-arterial pressure < 20 mm Hg

D. Measured patient urine output of 1 mL/kg per hour

E. Chest compression fraction < 80%

20. What is the first treatment priority for a patient who achieves ROSC?

A. Coronary reperfusion

B. Therapeutic hypothermia

C. Maintaining blood glucose < 200 mg/dL

D. Optimizing ventilation and oxygenation

E. Optimized hemodynamic goal

21. Choose an appropriate indication to stop or withhold resuscitation efforts.

a. Arrest not witnessed

b. Evidence of rigor mortis

c. Patient age greater than 85 years

d. No return of spontaneous circulation after 10 minutes of CPR

e. Initial rhythm is PEA

22. A team leader orders 1 mg of epinephrine, and a team member verbally acknowledges when the medication is administered. What element of effective resuscitation team dynamics does this represent?

A. Clear messages
B. Knowing one's limitations
C. Closed-loop communication
D. Clear roles and responsibilities
E. None of above

23. Which drug and dose are recommended for the management of a patient in refractory ventricular fibrillation?
A. $MgSO_4$ 2 gm
B. Amiodarone 300 mg
C. Adrenaline 1 mg
D. Lidocaine 1-1.5 mg/kg
E. B and D

24. What is the recommended target temperature and duration of target temperature management?
A. 32°C to 34 ^{o}C, at least 12 hours
B. 32°C to 34 ^{o}C, at least 24 hours
C. 32°C to 36 ^{o}C, at least 12 hours
D. 32°C to 36 ^{o}C, at least 24 hours
E. 34°C to 36 ^{o}C, at least 12 hours

25. Emergency medical responders are unable to obtain a peripheral IV for a patient in cardiac arrest. What is the next most preferred route for drug administration?
A. Intraosseous (IO)
B. Endotracheal (ET)
C. Intramuscular (IM)
D. Central venous access
E. Intradermal (ID)

26. Which situation best describes return of spontaneous circulation?
A. Spontaneous arterial pressure wave with A-line monitors
B. Abrupt sustained increase in $PETCO_2$ > 20 mmHg
C. Present of pulse with blood pressure
D. A and C
E. A, B and C

27. You are working in an emergency department. Your patient is a 69-year-old male who is being treated for chest pain. He has an IV in place and is on a bedside monitor. Diagnostic tests are complete and he is awaiting the arrival of the cardiac catheterization team. You go into his room to check on him and he suddenly loses consciousness. **You look up at the monitor and see ventricular fibrillation.** You are not sure whether you feel a pulse or not. You should immediately

    A. Have another nurse to double check the presence of a pulse
    B. Precordial thumb
    C. Immediate defibrillate at 200 J
    D. CPR 5 cycle
    E. Ventilate using the BVM

28. The most important intervention with witnessed sudden cardiac arrest is?

    A. effective chest compressions
    B. early defibrillation
    C. early activation of EMS
    D. rapid use of resuscitation drug
    E. Advance airway management

29. The H's and T's that are possible causes of PEA include all the following **EXCEPT**:

    A. hypovolemia, toxins, thrombosis
    B. hydrogen ion, hypokalemia, tamponade
    C. hypothermia, tension pneumothorax, hydrogen ion (acidosis)
    D. hydrogen ion, Hypothermia
    E. thrombocytopenia, hypoglycemia, hemophilia

30. The two most important aspects to treating PEA are:

    A. Provide effective CPR and check pulses regularly
    B. Provide effective CPR and timely transcutaneous pacing of the patient.
    C. Provide effective CPR and promptly use core drugs
    D. Provide effective CPR and correct the underlying cause of the rhythm.
    E. Provide effective CPR and immediate defibrillation
