# Supplementary material for: Precourse Preparation Using a Serious Smartphone Game on Advanced Life Support Knowledge and Skills: Randomized Controlled Trial
Source: J Med Internet Res. 2020 Mar 9;22(3):e16987. doi: 10.2196/16987 (PMC7091031; doi:10.2196/16987)
Supplement: Multimedia Appendix 2 [file jmir_v22i3e16987_app2.pdf]

Multimedia appendix 2: Skill test (A, B, C)

name...result...

A: One person ACLS management (Mega Code Essential Skill) in 5 minutes

stable SVT	Unstable SVT after reassessment
step of ABCs assessment	ECG interpretation: SVT
IV access, ECG monitor (lead II)	cardiovascular assess (pulse, BP, sign of shock)
ECG interpretation: SVT	step of ABCs assessment
cardiovascular assess (pulse, BP, sign of shock)	consider sedative drug or analgesics
consider carotid massage	proper cardioversion (step, dose, safety)
adenosine with proper administration technique	
Sign	

B: One person ACLS management (Mega Code Essential Skill) in 5 minutes

Unstable SVT	witness VF after first cardioversion
Unstable SVT	Call for help
step of ABCs assessment	Immediate DF
IV access, ECG monitor (lead II)	Defibrillation (step, dose, safety)
ECG interpretation: SVT	immediate CPR
cardiovascular assess (pulse, BP, sign of shock)	step of CPR
consider sedative drug or analgesics	proper medication
proper cardioversion (step, dose, safety)	
Sign	

C: One person ACLS management (Mega Code Essential Skill) in 5 minutes

Unstable Bradycardia	PEA
step of ABCs assessment	cardiovascular assess (pulse, BP, sign of shock)
IV access, ECG monitor (lead II)	Call for help
ECG interpretation	immediate CPR
cardiovascular assess (pulse, BP, sign of shock)	step of CPR
atropine 0.5 mg IV	proper medication
consider sedative drug or analgesics	6T6H
proper pacing (step, safety)	
Sign	
