# Supplementary material for: Precourse Preparation Using a Serious Smartphone Game on Advanced Life Support Knowledge and Skills: Randomized Controlled Trial
Source: J Med Internet Res. 2020 Mar 9;22(3):e16987. doi: 10.2196/16987 (PMC7091031; doi:10.2196/16987)
Supplement: Multimedia Appendix 3 [file jmir_v22i3e16987_app3.pdf]

Multimedia appendix 3: Questionnaire of Intervention group (Adapt from original Questionnaire in Thai language)

General information

1. Age ☐ years

2. Sex ☐ Male ☐ Female

3. CPR member experienced
   ☐ case/month ☐ < 1 case /month
   ☐< 1 case / 3 month ☐ never

   CPR leader experienced
   ☐ case/month ☐ < 1 case /month
   ☐< 1 case / 3 month ☐ never

4. CPR confident before CPR training course
   1 2 3 4 5 6 7 8 9 10
   least ⟷ the most

5. CPR confident after CPR training course
   1 2 3 4 5 6 7 8 9 10
   least ⟷ the most

6. ALS training course satisfaction
   0 1 2 3 4 5 6 7 8 9 10
   least ⟷ the most

7. Overall satisfaction with Resus Days application.
   0 1 2 3 4 5 6 7 8 9 10
   least ⟷ the most

8. Resus Days application help to improve CPR knowledge?
   1 2 3 4 5 6 7 8 9 10
   Totally agree ⟷ Totally disagree

   If agree, please give a reason
   ………………………………………………………………………………..
   If dis agree, please give a reason
   ……………………………………………………………………………….

9. Any suggestions for improving the game?
   ……………………………………………………………………………….

10. Frequency of playing games online by smart phone?

☐ Every day ☐ Every 2-3 day ☐ every week ☐ < 1 time/month ☐ Never

Supplementary materials-4.2
Questionnaire of control group (adapt from original Questionnaire in Thai language)

General information

1. Age ☐ years

2. Sex ☐ Male ☐ Female

3. CPR member experienced
   ☐ case/month ☐ < 1 case /month
   ☐< 1 case / 3 month ☐ never

   CPR leader experienced
   ☐ case/month ☐ < 1 case /month
   ☐< 1 case / 3 month ☐ never

4. CPR confident before CPR training course
   1 2 3 4 5 6 7 8 9 10
   least ⟷ the most

5. CPR confident after CPR training course
   1 2 3 4 5 6 7 8 9 10
   least ⟷ the most

6. ALS training course satisfaction
   1 1 2 3 4 5 6 7 8 9 10
   least ⟷ the most
