# Supplementary material for: Precourse Preparation Using a Serious Smartphone Game on Advanced Life Support Knowledge and Skills: Randomized Controlled Trial
Source: J Med Internet Res. 2020 Mar 9;22(3):e16987. doi: 10.2196/16987 (PMC7091031; doi:10.2196/16987)
Supplement: Multimedia Appendix 4 [file jmir_v22i3e16987_app4.pdf]

Multimedia appendix4: Resus Days screenshots

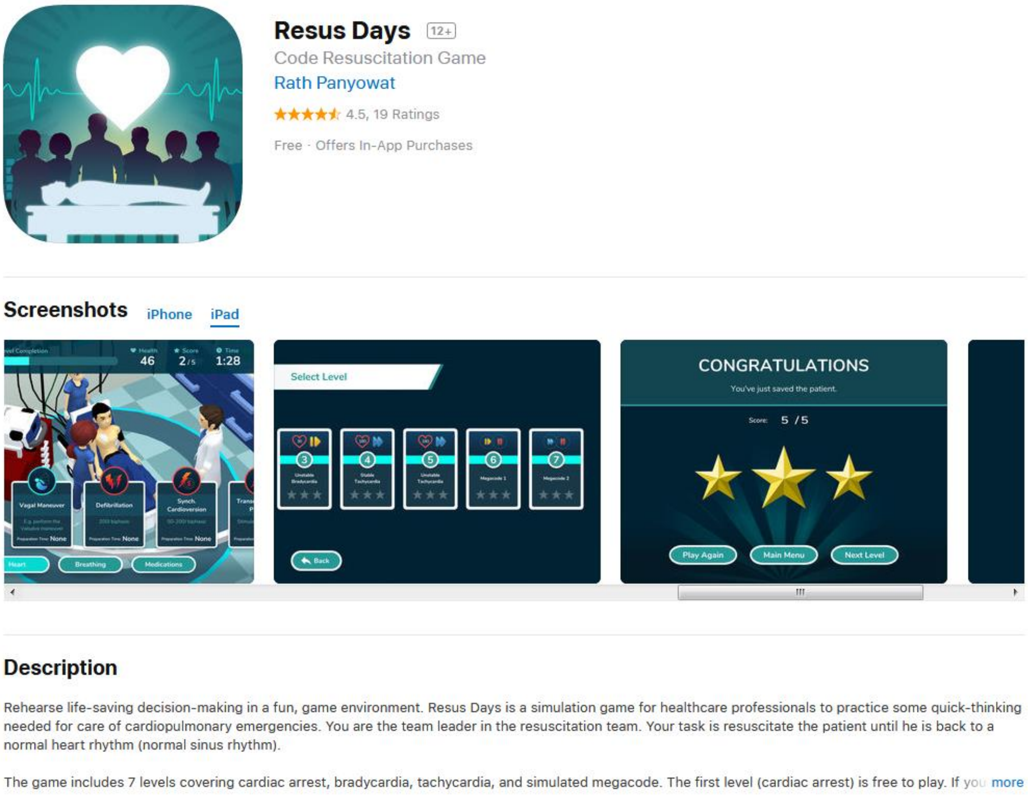

Screenshots

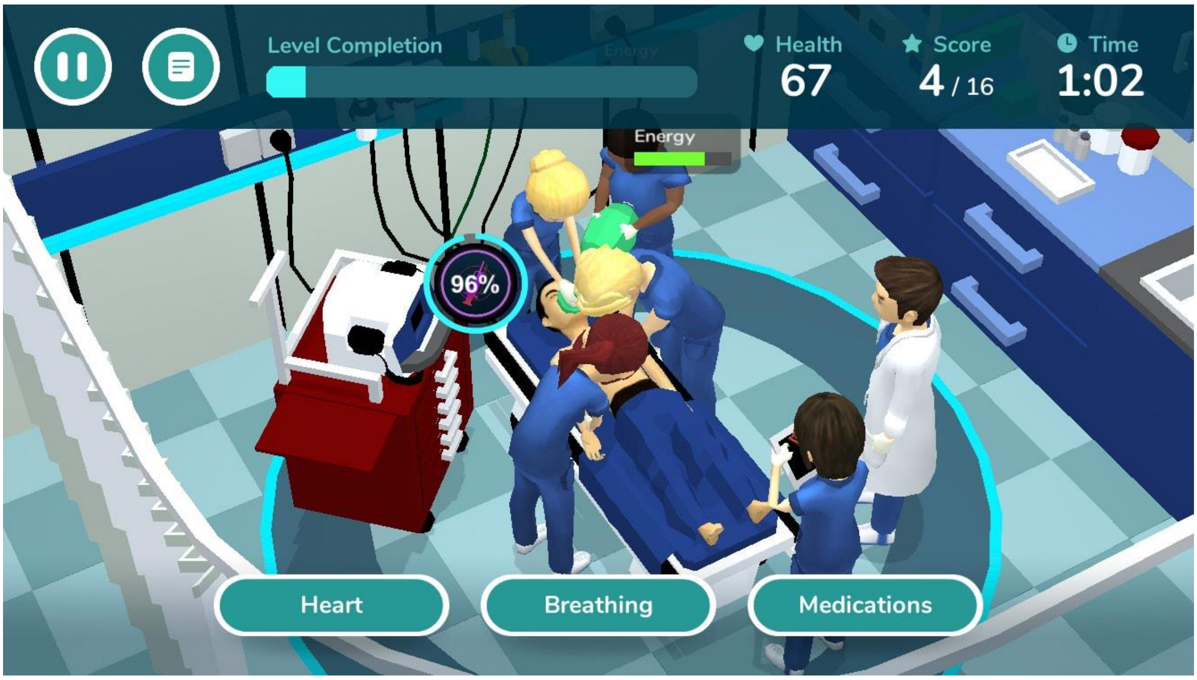

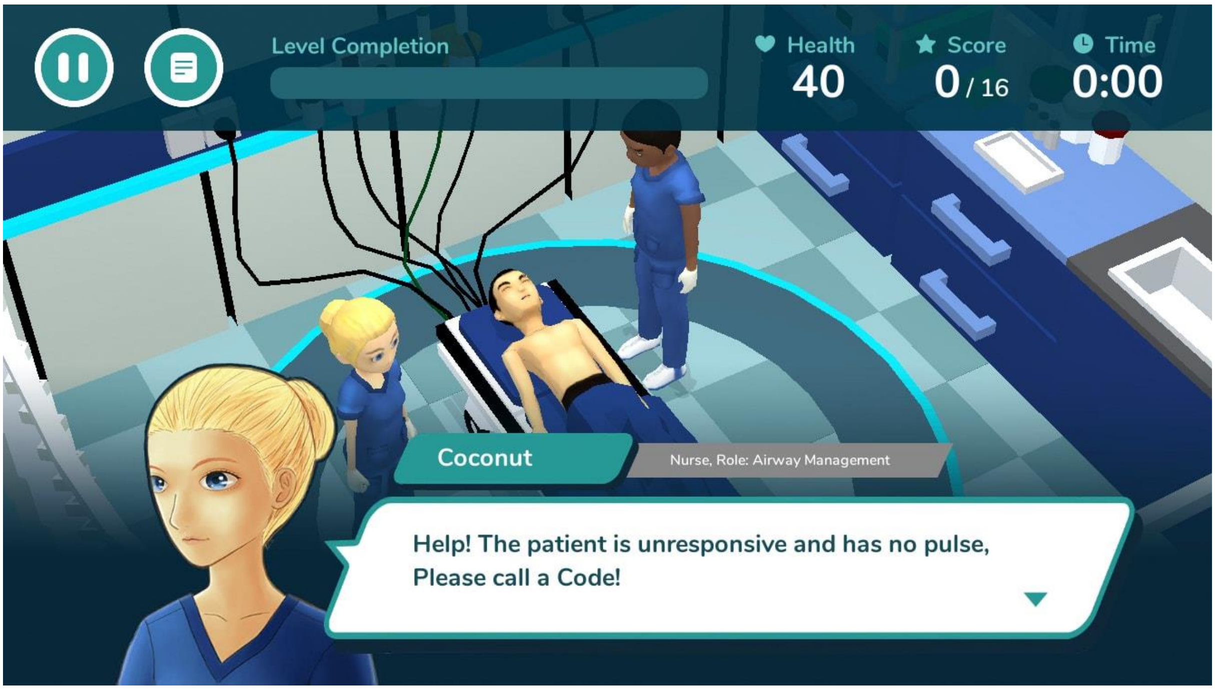

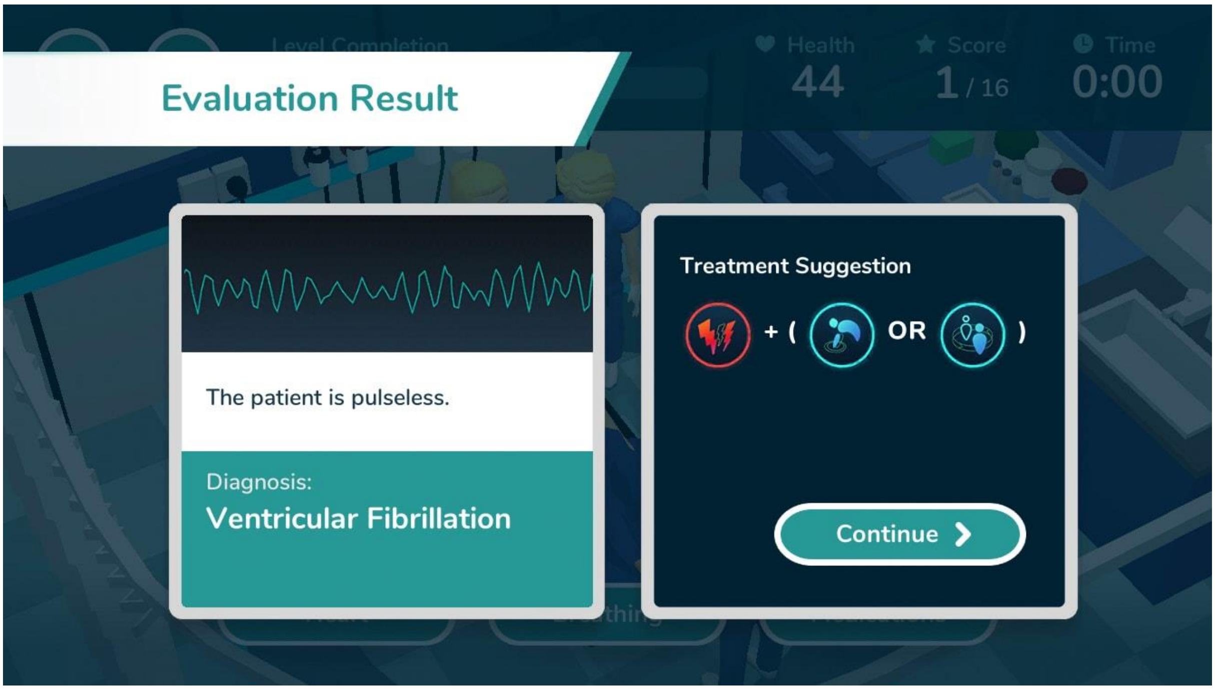
Evaluation Result
Health
44
Score
1 / 16
Time
0:00
The patient is pulseless.
Diagnosis:
Ventricular Fibrillation
Treatment Suggestion
+ (OR)
Continue

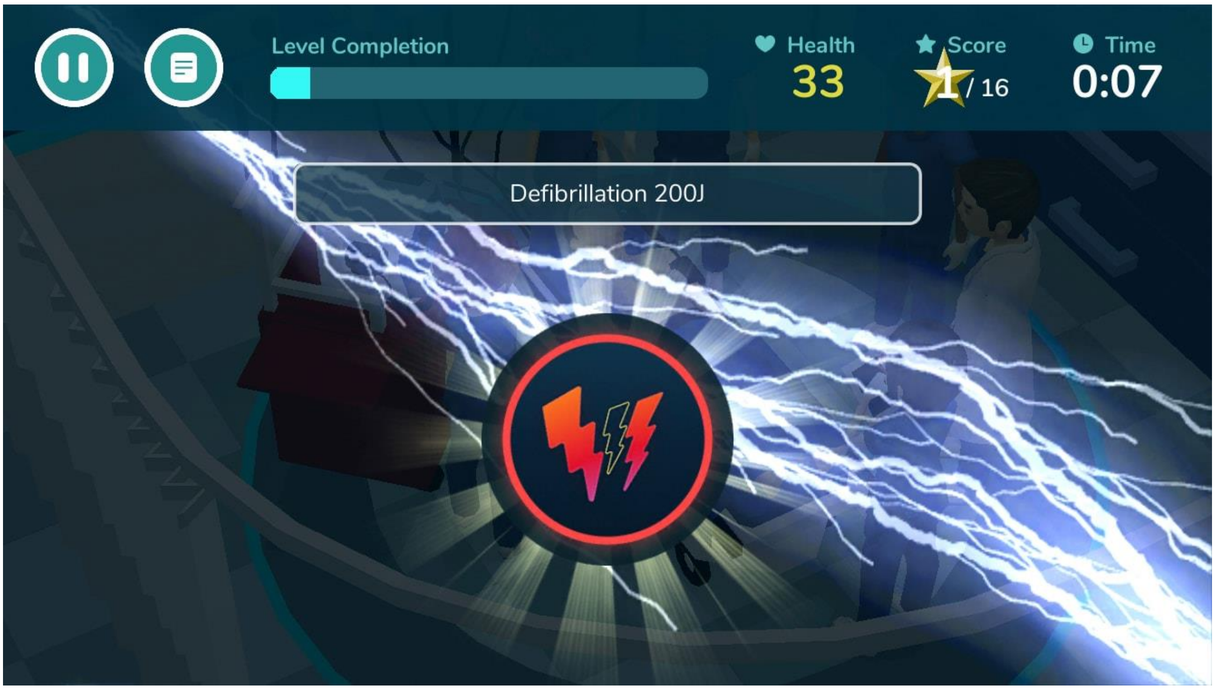
Level Completion
Health
33
Score
1 / 16
Time
0:07
Defibrillation 200J

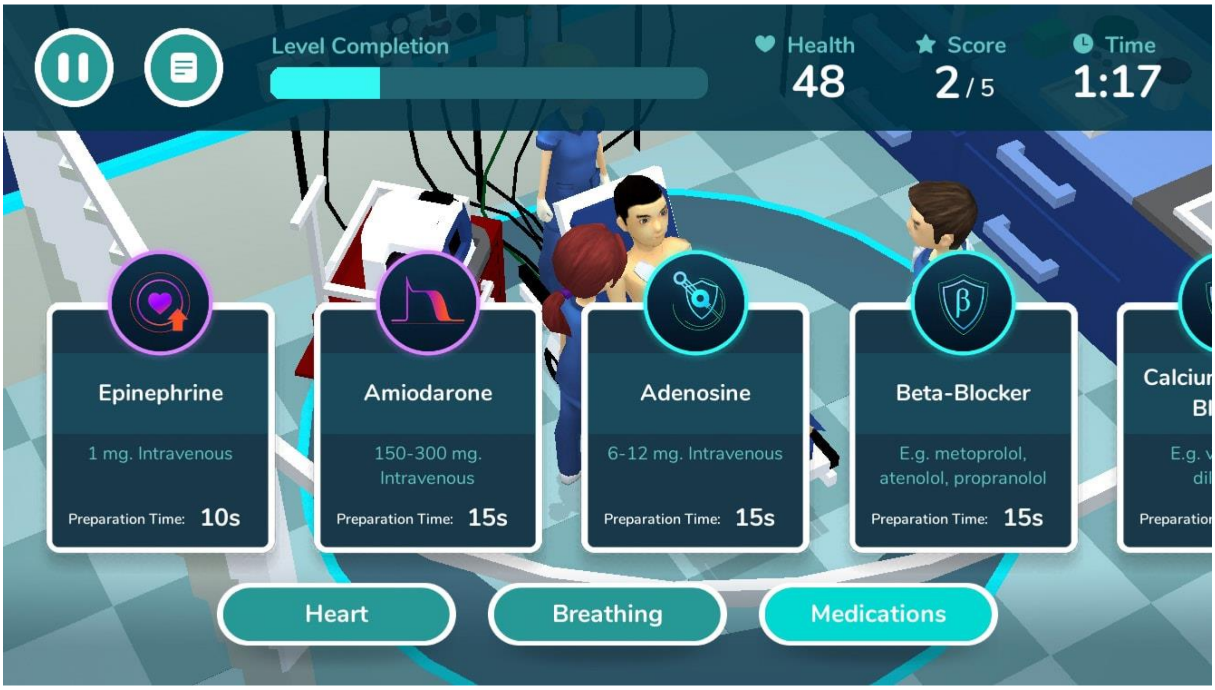
Level Completion
Health
48
Score
2 / 5
Time
1:17
Epinephrine
1 mg. Intravenous
Preparation Time: 10s
Amiodarone
150-300 mg. Intravenous
Preparation Time: 15s
Adenosine
6-12 mg. Intravenous
Preparation Time: 15s
Beta-Blocker
E.g. metoprolol, atenolol, propranolol
Preparation Time: 15s
Heart
Breathing
Medications

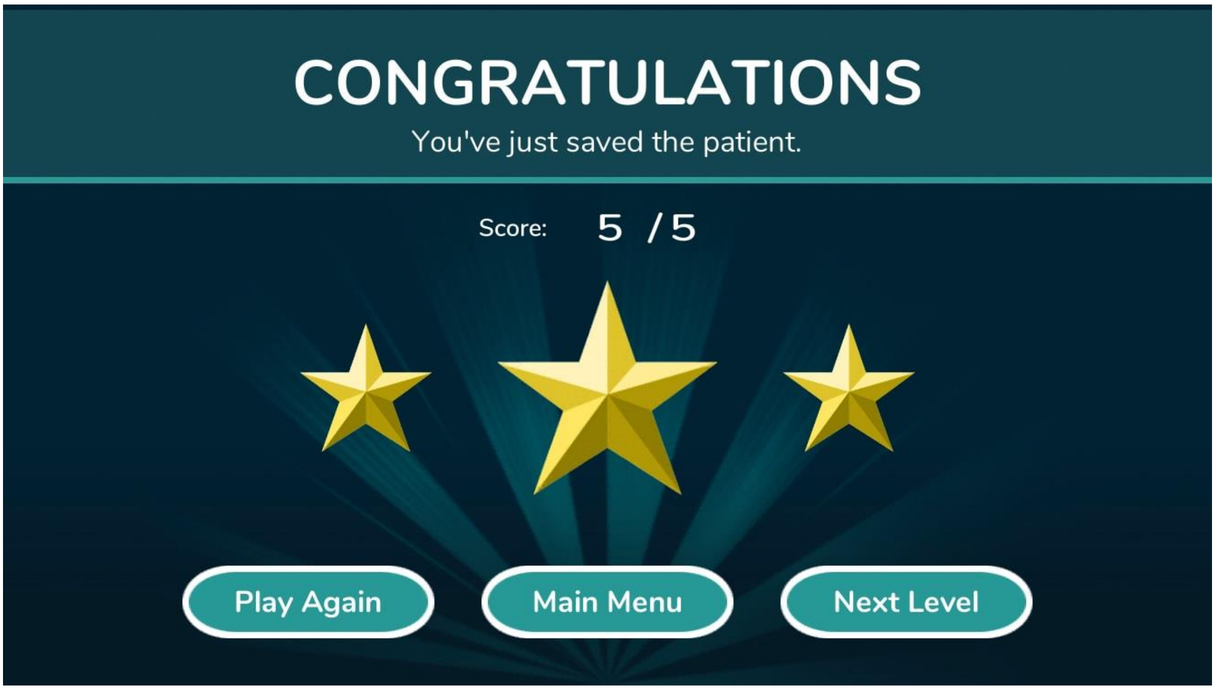
CONGRATULATIONS
You've just saved the patient.
Score: 5 / 5
Play Again
Main Menu
Next Level

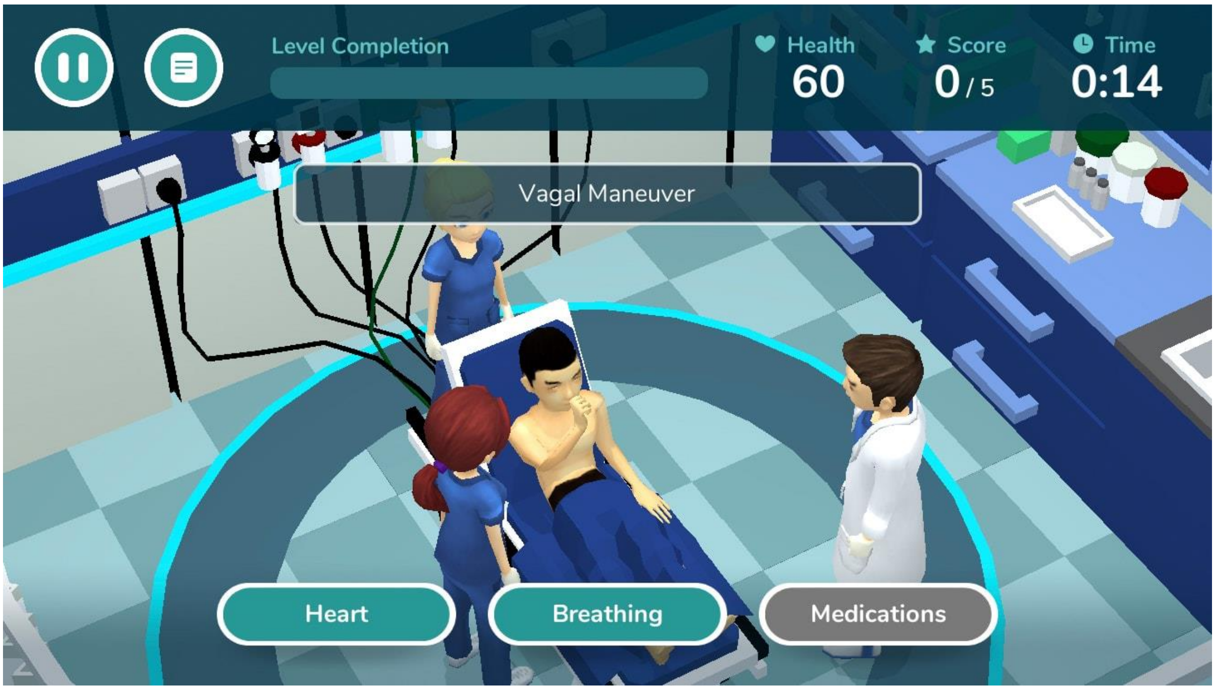
Level Completion
Health
60
Score
0 / 5
Time
0:14
Vagal Maneuver
Heart
Breathing
Medications

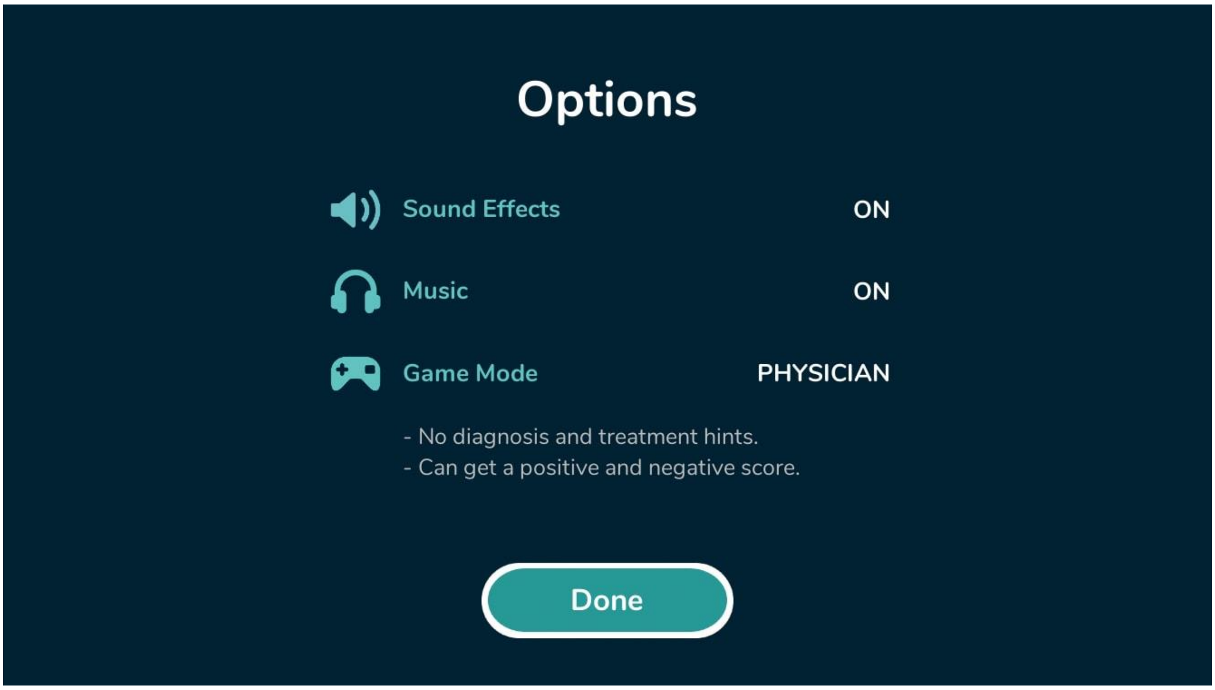
Options
Sound Effects
ON
Music
ON
Game Mode
PHYSICIAN
- No diagnosis and treatment hints.
- Can get a positive and negative score.
Done
